# Supplementary material for: A Likelihood Approach for Real-Time Calibration of Stochastic Compartmental Epidemic Models
Source: PLoS Comput Biol. 2017 Jan 17;13(1):e1005257. doi: 10.1371/journal.pcbi.1005257 (PMC5240920; doi:10.1371/journal.pcbi.1005257)
Supplement: S1 File — (TAR.GZ) [file pcbi.1005257.s014.tar.gz › HSPH_Online-SI-Revision/PF08/n10-severe/n10-severe_PF_table-4.pdf]

1	R0	{1.68952, 11.8402}	{1.61315, 19.2222}	{{1.51572, 18.4463}, {1.72015, 20.}}	{5.59495, 62.3469}	inf	719.	840.402	{652.192, 1017.82}	20.0884	0.0773163	newly	473.	315.85	{250., 394.}	33.2241	0.180752	Reff	1.34131	1.23695	{1.14666, 1.29764}	7.79821	ar	6872.	7490.8	{6857., 8053.}	9.52008
2	R0	{1.71739, 9.62661}	{1.61704, 19.0082}	{{1.48917, 14.8699}, {1.79146, 20.}}	{8.41525, 97.4548}	inf	504.	711.654	{553.909, 912.09}	41.2012	0.14168	newly	426.	276.03	{200., 361.}	35.3357	0.199065	Reff	1.47352	1.34054	{1.23398, 1.43596}	9.29126	ar	6874.	7552.83	{6854., 8284.}	10.271
3	R0	{1.48936, 6.23975}	{1.76895, 17.8291}	{{1.50138, 12.6667}, {2.06138, 20.}}	{19.5166, 185.735}	inf	371.	727.188	{418.989, 1016.97}	96.0096	0.275408	newly	491.	309.73	{224., 392.}	37.1955	0.213111	Reff	1.29008	1.46341	{1.2276, 1.66684}	15.3303	ar	5666.	7833.6	{6590., 8795.}	38.2563
4	R0	{1.62394, 7.51135}	{1.56386, 19.7275}	{{1.51985, 19.2629}, {1.6302, 20.}}	{3.87775, 162.636}	inf	401.	732.942	{636.603, 861.023}	82.7786	0.259163	newly	435.	267.91	{233., 311.}	38.4115	0.213331	Reff	1.42192	1.21459	{1.18745, 1.23687}	14.5813	ar	6417.	7269.16	{6936., 7613.}	13.2797
5	R0	{1.53632, 8.57652}	{1.58759, 19.6667}	{{1.50146, 18.9355}, {1.67318, 20.}}	{4.18522, 129.308}	inf	485.	828.484	{731.727, 951.311}	70.8215	0.229725	newly	460.	305.13	{264., 354.}	33.6674	0.181341	Reff	1.29543	1.21634	{1.18546, 1.24741}	6.11292	ar	6260.	7568.2	{7223., 7966.}	20.8978
6	R0	{1.47166, 5.7434}	{1.56594, 19.4666}	{{1.50033, 18.7198}, {1.62871, 20.}}	{6.4404, 238.939}	inf	232.	679.302	{575.893, 827.114}	192.803	0.462327	newly	328.	253.33	{212., 309.}	23.1677	0.118637	Reff	1.32906	1.25277	{1.21512, 1.29755}	5.76312	ar	5650.	7131.18	{6763., 7555.}	26.2156
7	R0	{1.48179, 8.87369}	{1.63582, 19.0164}	{{1.49733, 14.4135}, {1.83635, 20.}}	{10.6938, 114.301}	inf	510.	959.843	{685.114, 1251.65}	88.2044	0.264869	newly	397.	363.42	{275., 453.}	15.9446	0.0753063	Reff	1.14379	1.17194	{1.06003, 1.23278}	4.92384	ar	5233.	7606.31	{6921., 8364.}	45.3528
8	R0	{1.53669, 6.47807}	{1.73862, 18.2428}	{{1.48263, 13.5889}, {2.07147, 20.}}	{14.3503, 181.608}	inf	392.	787.058	{560.561, 1011.44}	100.788	0.290032	newly	477.	323.8	{232., 405.}	32.2306	0.177578	Reff	1.32417	1.3903	{1.24543, 1.51902}	8.49395	ar	5934.	7635.81	{6393., 8623.}	28.679
9	R0	{1.57227, 6.20989}	{1.71488, 17.5032}	{{1.35421, 12.3168}, {2.10326, 20.}}	{16.4082, 181.86}	inf	215.	379.692	{218.228, 619.983}	77.8897	0.21719	newly	311.	171.51	{93., 292.}	46.8778	0.302951	Reff	1.49381	1.55396	{1.26828, 1.864}	13.9793	ar	6136.	7123.87	{5281., 8624.}	21.9917
10	R0	{1.54936, 6.50938}	{2.08454, 16.1162}	{{1.46839, 10.72}, {2.61377, 20.}}	{36.8375, 147.584}	inf	423.	842.695	{385.642, 1240.22}	103.54	0.285086	newly	555.	417.68	{235., 546.}	26.6883	0.152007	Reff	1.36483	1.72143	{1.26989, 2.15575}	28.6044	ar	6275.	8296.78	{6249., 9481.}	34.212
11	R0	{1.42179, 3.18601}	{1.72601, 17.9993}	{{1.46663, 13.1178}, {2.06197, 20.}}	{21.482, 464.949}	inf	124.	478.321	{310.741, 622.965}	285.743	0.565716	newly	375.	208.24	{129., 294.}	45.136	0.278258	Reff	1.33549	1.55708	{1.31496, 1.81364}	17.2767	ar	5322.	7663.38	{6423., 8866.}	43.9944
12	R0	{1.48035, 6.46183}	{1.71707, 18.0904}	{{1.44846, 13.3725}, {2.07484, 20.}}	{16.8225, 179.958}	inf	191.	477.51	{288.869, 670.424}	150.005	0.378104	newly	266.	207.01	{130., 281.}	27.5677	0.150968	Reff	1.39049	1.54004	{1.36426, 1.79257}	12.0606	ar	5793.	7573.19	{6385., 8795.}	30.73
13	R0	{1.75755, 8.53854}	{1.69706, 19.3177}	{{1.50584, 16.4795}, {1.9065, 20.}}	{9.05394, 126.241}	inf	456.	678.024	{468.838, 914.846}	49.0278	0.158662	newly	444.	263.67	{183., 355.}	40.6149	0.241259	Reff	1.57195	1.4436	{1.32221, 1.55321}	8.62582	ar	6832.	7806.37	{6912., 8654.}	14.7542
14	R0	{1.47355, 4.89451}	{1.79568, 17.7483}	{{1.48571, 14.0457}, {2.13791, 20.}}	{22.2371, 262.617}	inf	339.	743.613	{448.643, 1086.57}	119.686	0.319182	newly	540.	319.39	{205., 458.}	41.0685	0.248274	Reff	1.31367	1.49851	{1.28469, 1.68486}	15.4262	ar	5710.	7892.42	{6503., 9008.}	38.221
15	R0	{1.62071, 5.98665}	{1.97377, 16.4171}	{{1.41137, 9.35196}, {2.62213, 20.}}	{28.9173, 174.228}	inf	269.	508.048	{190.009, 889.55}	100.433	0.265591	newly	451.	254.54	{87., 416.}	46.5987	0.319383	Reff	1.51245	1.77762	{1.31864, 2.38331}	24.311	ar	6635.	7894.03	{5914., 9617.}	24.4942
16	R0	{1.67268, 7.7256}	{2.17835, 17.5532}	{{1.67227, 12.0761}, {2.62368, 20.}}	{31.299, 127.209}	inf	657.	1325.42	{788.754, 1866.94}	102.652	0.288322	newly	678.	583.29	{432., 733.}	18.9867	0.0957702	Reff	1.34935	1.60266	{1.34812, 1.82076}	20.0144	ar	6947.	9073.07	{7603., 9907.}	30.9502
17	R0	{1.67805, 11.1417}	{1.71481, 17.4839}	{{1.46176, 12.5839}, {2.07189, 20.}}	{11.6615, 57.2186}	inf	455.	615.856	{387.738, 883.323}	41.8141	0.140721	newly	326.	268.66	{185., 361.}	22.8221	0.118873	Reff	1.48021	1.4697	{1.26009, 1.68404}	9.29558	ar	6625.	7553.83	{6235., 8800.}	16.7567
18	R0	{1.66743, 9.99046}	{1.63342, 18.4756}	{{1.45576, 13.5871}, {1.87377, 20.}}	{8.87824, 84.9321}	inf	487.	657.112	{508.994, 913.736}	36.8556	0.125136	newly	415.	265.05	{196., 352.}	36.5036	0.208838	Reff	1.42932	1.34674	{1.23606, 1.48866}	7.95493	ar	6899.	7224.07	{6305., 8269.}	8.87563
19	R0	{1.70787, 9.85478}	{1.58256, 19.4833}	{{1.50848, 18.6603}, {1.63816, 20.}}	{7.41817, 97.704}	inf	650.	837.366	{703.198, 943.76}	29.0055	0.107338	newly	499.	309.43	{260., 354.}	37.99	0.211474	Reff	1.37449	1.20352	{1.16186, 1.25747}	12.4389	ar	6867.	7499.47	{7014., 7865.}	9.21086
20	R0	{1.53809, 10.5848}	{1.56078, 19.77}	{{1.50388, 19.2343}, {1.62075, 20.}}	{2.59061, 86.7769}	inf	420.	795.783	{727.942, 884.977}	89.4722	0.276275	newly	300.	292.75	{264., 329.}	7.39	0.0327514	Reff	1.34152	1.22585	{1.19744, 1.25971}	8.62276	ar	6108.	7465.61	{7164., 7748.}	22.2268
21	R0	{1.59925, 6.74479}	{1.99363, 15.9366}	{{1.45117, 10.0795}, {2.56078, 20.}}	{28.6836, 136.346}	inf	387.	734.168	{325.98, 1169.35}	95.7171	0.264371	newly	488.	369.52	{188., 523.}	29.6475	0.17112	Reff	1.42989	1.68554	{1.27717, 2.14759}	22.1683	ar	6411.	8087.01	{6091., 9445.}	28.0276
22	R0	{1.38779, 4.40977}	{1.68206, 18.0425}	{{1.42726, 13.5063}, {1.99379, 20.}}	{21.3017, 309.148}	inf	183.	548.616	{342.871, 744.039}	199.79	0.456178	newly	337.	231.59	{157., 308.}	33.273	0.187711	Reff	1.26761	1.47727	{1.27214, 1.6814}	16.9999	ar	5154.	7473.97	{6248., 8544.}	45.013
23	R0	{1.53657, 7.50352}	{1.79254, 18.4793}	{{1.47618, 14.399}, {2.09677, 20.}}	{18.1486, 146.275}	inf	441.	791.052	{541.048, 995.331}	79.9995	0.243169	newly	429.	324.96	{236., 422.}	26.0047	0.139295	Reff	1.31868	1.45197	{1.22462, 1.60972}	12.4618	ar	5928.	7878.71	{6516., 8810.}	32.9067
24	R0	{1.71131, 6.24526}	{1.75991, 19.2639}	{{1.53738, 17.8496}, {2.0615, 20.}}	{9.5883, 208.457}	inf	217.	581.098	{420.633, 748.865}	167.787	0.416358	newly	358.	230.11	{168., 298.}	36.0363	0.204752	Reff	1.60743	1.55604	{1.39156, 1.78935}	8.80813	ar	6924.	8046.26	{7152., 9094.}	16.6502
25	R0	{1.4412, 3.07729}	{1.69753, 18.0026}	{{1.36859, 12.9645}, {2.10794, 20.}}	{19.8595, 485.016}	inf	56.	274.034	{165.302, 435.297}	389.347	0.661251	newly	207.	121.61	{69., 188.}	42.5072	0.266233	Reff	1.41454	1.60509	{1.30244, 1.91921}	17.1071	ar	5810.	7106.94	{5424., 8654.}	25.4788
26	R0	{1.4159, 4.93858}	{1.89898, 16.882}	{{1.40686, 12.2351}, {2.44896, 20.}}	{34.8589, 241.839}	inf	225.	627.229	{320.028, 984.856}	179.131	0.403936	newly	397.	300.92	{141., 472.}	34.3375	0.20262	Reff	1.30079	1.64113	{1.26496, 2.01469}	27.4425	ar	5255.	7801.28	{5933., 9224.}	48.6885
27	R0	{1.57465, 7.05414}	{1.82242, 16.7854}	{{1.39681, 12.331}, {2.31689, 20.}}	{22.2348, 138.186}	inf	216.	401.632	{205.644, 653.589}	88.1334	0.231045	newly	292.	196.36	{91., 327.}	42.6918	0.259672	Reff	1.4934	1.65777	{1.28842, 2.1129}	19.4202	ar	6359.	7440.42	{5598., 9177.}	22.5278
28	R0	{1.41422, 4.62402}	{1.84097, 17.489}	{{1.46495, 12.4344}, {2.31385, 20.}}	{30.3287, 278.221}	inf	138.	469.429	{270.915, 757.239}	240.166	0.494833	newly	285.	216.99	{119., 357.}	35.9193	0.200819	Reff	1.35298	1.67345	{1.34431, 1.99412}	24.3376	ar	5485.	7910.2	{6437., 9269.}	44.2151
29	R0	{1.41901, 3.63017}	{1.98839, 17.2405}	{{1.43021, 12.2242}, {2.56816, 20.}}	{40.8706, 374.923}	inf	127.	417.693	{166.785, 802.758}	228.892	0.447339	newly	382.	202.06	{84., 343.}	50.0524	0.353858	Reff	1.3631	1.82731	{1.35877, 2.3474}	34.7828	ar	5979.	8030.51	{6054., 9476.}	36.252
30	R0	{1.60719, 4.72984}	{1.89061, 16.7815}	{{1.41586, 11.0814}, {2.44533, 20.}}	{23.7687, 254.802}	inf	120.	346.918	{170.478, 591.537}	189.099	0.409055	newly	274.	173.	{76., 279.}	43.4745	0.276609	Reff	1.56171	1.76248	{1.32453, 2.21069}	20.3665	ar	6479.	7669.91	{5709., 9277.}	23.2531
31	R0	{1.38175, 6.26468}	{1.88406, 16.3397}	{{1.38289, 10.9622}, {2.29174, 20.}}	{36.9127, 160.823}	inf	357.	770.639	{379.502, 1125.32}	117.649	0.305745	newly	432.	366.62	{207., 537.}	26.125	0.143428	Reff	1.2035	1.54406	{1.20021, 1.84782}	28.8962	ar	5397.	7780.42	{5452., 9032.}	44.6811
32	R0	{1.4947, 8.8847}	{1.58469, 19.4732}	{{1.48635, 18.9648}, {1.67061, 20.}}	{6.35124, 119.177}	inf	462.	860.018	{713.225, 1046.52}	86.151	0.264935	newly	361.	315.52	{264., 374.}	15.1191	0.0729194	Reff	1.18634	1.18436	{1.13208, 1.22503}	2.48047	ar	5407.	7429.97	{6961., 7932.}	37.4139
33	R0	{1.55891, 8.28978}	{1.54646, 19.5356}	{{1.46401, 18.8275}, {1.61713, 20.}}	{3.24739, 135.658}	inf	446.	759.744	{634.806, 918.252}	70.3462	0.227169	newly	409.	280.62	{237., 346.}	31.6479	0.169426	Reff	1.30107	1.19198	{1.159, 1.22316}	8.38462	ar	6239.	7141.4	{6710., 7567.}	14.4639
34	R0	{1.60764, 10.4729}	{1.61587, 19.3417}	{{1.49923, 18.6252}, {1.71366, 20.}}	{4.3083, 84.683}	inf	658.	934.583	{719.111, 1166.01}	42.4411	0.1467	newly	455.	348.07	{282., 431.}	24.3978	0.126287	Reff	1.23097	1.18395	{1.13169, 1.2428}	4.39492	ar	6495.	7558.67	{6941., 7994.}	16.5541
35	R0	{1.48527, 5.74485}	{1.75205, 17.8669}	{{1.50033, 13.7283}, {2.10252, 20.}}	{18.4816, 211.007}	inf	223.	530.838	{339.265, 743.499}	138.044	0.356739	newly	365.	233.13	{147., 324.}	37.7945	0.221	Reff	1.37566	1.55242	{1.31724, 1.79803}	14.4255	ar	5811.	7744.67	{6554., 8931.}	33.3459
36	R0	{1.61457, 9.93364}	{1.68961, 19.0887}	{{1.49275, 14.8671}, {1.87971, 20.}}	{8.41032, 92.1623}	inf	466.	788.071	{552.386, 1030.06}	69.1139	0.215041	newly	363.	307.72	{217., 395.}	19.73	0.101016	Reff	1.39273	1.37886	{1.24897, 1.50662}	5.29259	ar	6338.	7846.73	{6945., 8703.}	23.8045
37	R0	{1.47831, 7.08139}	{1.81421, 18.1323}	{{1.4795, 13.2687}, {2.11772, 20.}}	{23.3149, 156.056}	inf	307.	701.213	{432.328, 1034.85}	128.408	0.337824	newly	351.	301.27	{193., 422.}	23.9801	0.121122	Reff	1.31939	1.53091	{1.30281, 1.72463}	17.4589	ar	5530.	7969.81	{6541., 8910.}	44.1535
38	R0	{1.73445, 9.8858}	{1.71564, 17.6145}	{{1.46144, 13.2019}, {2.07607, 20.}}	{11.1737, 78.7168}	inf	459.	593.167	{375.266, 891.786}	37.9568	0.132023	newly	402.	258.26	{160., 357.}	36.7811	0.215147	Reff	1.53534	1.4923	{1.25465, 1.7305}	9.31318	ar	6842.	7621.89	{6412., 8909.}	14.6479
39	R0	{1.66621, 11.4824}	{1.64895, 19.1203}	{{1.4798, 17.7929}, {1.86104, 20.}}	{6.91614, 66.5179}	inf	585.	829.799	{604.136, 1125.28}	42.2677	0.141871	newly	373.	318.48	{231., 427.}	20.8579	0.104692	Reff	1.36629	1.30135	{1.18491, 1.39876}	6.10852	ar	6473.	7656.91	{6986., 8558.}	18.3573
40	R0	{1.60694, 9.95741}	{1.59892, 19.4423}	{{1.49689, 18.4939}, {1.69491, 20.}}	{4.1902, 95.2545}	inf	536.	744.777	{603.262, 894.464}	38.9741	0.137044	newly	419.	280.39	{222., 337.}	33.1671	0.181202	Reff	1.35481	1.29969	{1.21596, 1.3701}	4.80609	ar	6625.	7473.85	{6882., 8089.}	12.8928
41	R0	{1.55128, 4.71911}	{1.93684, 17.1058}	{{1.39888, 11.2125}, {2.59122, 20.}}	{29.296, 262.48}	inf	143.	372.877	{172.7, 668.079}	160.753	0.358769	newly	305.	182.96	{77., 313.}	45.9934	0.303251	Reff	1.48892	1.80277	{1.32735, 2.45297}	26.423	ar	6141.	7788.61	{5816., 9528.}	29.5129
42	R0	{1.60063, 6.81118}	{1.56615, 19.7099}	{{1.50535, 19.0538}, {1.62099, 20.}}	{2.97003, 189.376}	inf	336.	691.185	{595.018, 818.646}	105.71	0.310051	newly	454.	254.8	{218., 295.}	43.8767	0.25435	Reff	1.44265	1.26637	{1.2336, 1.29398}	12.2187	ar	6528.	7305.65	{6865., 7703.}	11.9125
43	R0	{1.56986, 5.44652}	{1.98976, 17.4963}	{{1.42947, 14.0212}, {2.58001, 20.}}	{30.5137, 221.238}	inf	263.	546.829	{248.609, 958.968}	111.185	0.284105	newly	451.	256.7	{111., 434.}	44.9446	0.299379	Reff	1.45887	1.7678	{1.33695, 2.22581}	25.062	ar	6312.	8003.48	{5968., 9509.}	29.3634
44	R0	{1.47523, 8.07785}	{1.65118, 19.2717}	{{1.4902, 18.2529}, {1.83681, 20.}}	{12.0519, 138.575}	inf	404.	739.331	{558.512, 947.205}	83.0026	0.25358	newly	403.	285.13	{215., 386.}	29.9628	0.161957	Reff	1.27976	1.36359	{1.26096, 1.453}	7.37641	ar	5692.	7658.31	{6926., 8381.}	34.5452
45	R0	{1.6417, 7.69429}	{1.82454, 17.8191}	{{1.50384, 13.6434}, {2.1105, 20.}}	{14.7119, 131.589}	inf	406.	707.561	{449.982, 928.75}	75.3311	0.228401	newly	444.	307.7	{207., 428.}	33.4955	0.186772	Reff	1.44486	1.52556	{1.31071, 1.71681}	10.327	ar	6735.	7966.11	{6475., 8878.}	19.8368
46	R0	{1.38865, 5.83473}	{1.68074, 17.5717}	{{1.44278, 12.7482}, {2.03574, 20.}}	{21.1121, 201.157}	inf	250.	634.131	{400.718, 851.864}	153.652	0.385222	newly	337.	271.92	{196., 334.}	24.0059	0.124429	Reff	1.23465	1.41606	{1.23138, 1.6332}	15.4347	ar	5233.	7478.77	{6116., 8766.}	42.9155
47	R0	{1.45289, 6.87903}	{1.74881, 16.7838}	{{1.3697, 11.1637}, {2.12374, 20.}}	{22.7777, 143.985}	inf	272.	668.209	{348.449, 1039.54}	146.543	0.355095	newly	303.	301.13	{178., 451.}	28.9274	0.131328	Reff	1.28537	1.44344	{1.17949, 1.66416}	15.9293	ar	5379.	7345.88	{5419., 8715.}	37.6728
48	R0	{1.69416, 9.4831}	{1.5946, 19.64}	{{1.49195, 18.8427}, {1.67451, 20.}}	{7.30359, 107.105}	inf	453.	647.331	{516.777, 794.977}	42.8987	0.148111	newly	442.	244.47	{198., 299.}	44.69	0.264248	Reff	1.51407	1.35601	{1.2994, 1.4468}	10.5555	ar	6925.	7381.79	{6828., 7924.}	7.41993
49	R0	{1.47843, 7.16286}	{1.70192, 17.7191}	{{1.44787, 13.1959}, {2.03299, 20.}}	{16.1364, 147.374}	inf	357.	651.232	{420.047, 885.309}	82.5679	0.244295	newly	403.	280.36	{203., 379.}	31.6774	0.176803	Reff	1.2988	1.43577	{1.24526, 1.6075}	12.6262	ar	5449.	7559.62	{6321., 8749.}	38.7341
50	R0	{1.68843, 5.72427}	{2.22529, 16.0151}	{{1.5107, 10.9954}, {3., 20.}}	{35.1119, 180.13}	inf	329.	725.598	{298.451, 1126.08}	125.403	0.325006	newly	521.	391.68	{197., 582.}	32.0115	0.192845	Reff	1.53732	1.88643	{1.33943, 2.40432}	27.0648	ar	6800.	8334.42	{6192., 9622.}	26.685
